# Northern Ohio Dental Association

**Published:** 1874-08

**Authors:** L. C. Kelsey


### NORTHERN OHIO DENTAL ASSOCIATION.

Put-in-Bay Island, June 9th, 1874, 10 A. M.

Meeting called to order by the President, Dr. S. P. Hildreth. On motion, L. C. Kelsey was appointed Secretary pro tem. Minutes of last meeting read and approved. The Committee on Order of Business submitted the following report.

SUBJECTS FOR DISCUSSION.

1. The care of the teeth during the period of dental development.

2. Separating teeth preparatory to filling.

3. Can fillings perfectly impervious to moisture be made with cohesive gold?

4. Dental ethics.

5. Miscellaneous.

On motion of L. C. Kelsey, Dr. W. C. Stewart of Elyria, was invited to sit with the Association, and participate in its deliberations.

Having organized, the association adjourned to meet at 2 o'clock P. M.

JUNE 9, 2 O'CLOCK P. M.

Meeting called to order by the President.

The following subject was then taken up for discussion.

Care of the teeth during the period of dental development.

Dr. Way considered this one of the most important subjects which could come before the Association.

Want of proper care of the teeth, during the period of dental development, is one of the principal causes of decay.

As a general thing, little care is bestowed upon the teeth until the eruption of the six year old molars. Many teeth might be saved if the parents would instruct their children to take proper care of them.

At the close of Dr. Way's remarks, Dr. Spelman, made a motion that all members of the profession, who are now present, or who may be present, not members of this Association, be invited to sit in the Association and take part in its deliberations, carried.

The further discussion of the subject was then resumed by Dr. C. Buffett, who remarked that the importance of proper care of the teeth, should be early impressed upon children by parents. The child is very imitative, it learns much by example. Whatever it sees the parent do it is very likely to do also. If the father and mother take good and constant care of their own teeth, the child will very readily be taught to observe habits of cleanliness in regard to its own teeth.

Dr. Knapp: One cause of decay is the fact that parents do not take sufficient care of the temporary teeth of their children. They are very apt to think the temporary teeth are of no account, and the sooner they are out of the way the better. Filling temporary teeth they would regard as a waste of money.

Dr. Stroud: Want of proper care of temporary teeth lays the foundation of much trouble with permanent teeth. Great care should be taken that the temporary teeth are not removed too soon, s ave them as long as possible by keeping them clean, and filling them when practicable.

Dr. Peebles would save the temporary teeth, from the fact that they are a great benefit to the child. Had treated and filled many troublesome temporary teeth with good results.

Dr. Spelman considered it bad practice to use arsenic in destroying tooth pulp, either in temporary or permanent teeth. He used carbolic acid, repeating the application until the pulp was wholly destroyed. Sometimes he used pepsin after the application of the carbolic acid. The use of arsenic tended to break down the cells and destroy the tooth. On motion further discussion of this subject was discontinued and the next in the order of business to-wit: "Separating teeth preparatory to filling was taken up for discussion."

Dr. Way: Since the introduction of the rubber-dam, it did not seem as necessary to separate teeth, preparatory to filling, either with the wedge or file, as formerly. As now found the cases were rare when it was necessary to use the file in preparing a tooth for filling.

Dr. Stroud: Would seldom use the file or wedge in filling teeth. Related his manner of proceeding in difficult cases. On motion, the Association adjourned to meet at 8 o'clock p. m.

JUNE 9, 8 O'CLOCK, P. M.

Meeting called to order by the President, Dr. Hildreth.

The subject of separating teeth preparatory to filling was further considered.

Dr. Peebles said he believed in using both the file and wedge, in preparing teeth for filling. Thought it the only way in many cases that good fillings could be made. Considered it difficult to observe and reach every point and space which needed attention without so doing. Freedom for the teeth was no objection and if the tooth after the use of the file, was properly smoothed and polished little if any danger might be apprehended.

Dr. Kelsey: The free use of the file or chisel in preparing teeth for filling is, in many cases, indispensable; decayed teeth with thin over laping edges could be successfully treated in no other way.

On motion, the Association adjourned to meet on Wednesday, June 10, at 9 o'clock, a. m.

MORNING SESSION, WEDNESDAY, JUNE 10, 9 O'CLOCK, A. M.

Meeting called to order by President Hildreth.

Dr. J. E. Robinson proposed Dr. F. W. Sage, of Sandusky, for membership in the association.

On motion, the application of F. W. Sage for membership was referred to the Examining Committee with instructions to report at once.

The Treasurer, J. E. Robinson, then made his report, which, on motion, was accepted.

Dr. C. Buffett, Corresponding Secretary, presented a bill for stationery and printing amounting to $6.61, and on motion, the Recording Secretary was instructed to draw an order on the Treasurer for the amount.

The Committee of Examiners, to whom was referred the application of F. W. Sage, reported favorably, and, on motion, the report was accepted, a ballot was then had, which resulted in the election of Dr. F. W. Sage to membership in the Association.

On motion, the Association then proceeded to the election of officers for the ensuing year, the result of which was as follows:

President, Dr. E. J. Waye, of Sandusky;
Vice-President, R. R. Peebles, of Cleveland;
Corresponding Secretary, C. Buffett, of Cleveland;
Recording Secretary, L. C. Kelsey, of Elyria;
Treasurer, J. E. Robinson, of Cleveland.

On motion, Drs. J. E. Robinson and C. Buffett were appointed a committee to make a selection of delegates to attend the American Dental Association, which meets at the city of Detroit, Michigan, on the fourth day of August, 1874.

On motion, the name of J. F. Galentine was dropped from the rolls of the Association.

The committee to whom was referred the selection of delegates to the American Dental Association, reported the following: Drs. J. E. Phelps, F. W. Sage, John Stephen, E. J. Waye, S. P. Hildreth, A. G. Price and D. R. Jennings. On motion, the report was accepted.

Dr. S. P. Hildreth then read a very interesting essay before the Association, entitled "The Education of the Dentist."

On motion, Dr. Hildreth was requested to furnish a copy of his essay for publication in the DENTAL REGISTER.

Drs. Waye and Sage, being called upon, read very interesting essays before the Asssociation on Dental Ethics.

The subject can fillings, perfectly impervious to moisture be made with cohesive gold was then taken up for discussion.

Dr. Hildreth, commenced the discussion by saying, that he was confident fillings perfectly impervious to moisture had, and could be made, with cohesive gold. Was surprised to see a statement in some journal that such a result with cohesive gold could not be obtained. He had not experimented upon it, but others in the association had and the result of their experiments, showed to him very conclusively that fillings perfectly impervious to moisture could be made with cohesive gold.

Dr. Waye, presented several teeth which he had filled and experimented upon, the result of which showed conclusively to himself and others that fillings could be made with cohesive gold perfectly impervious to moisture.

Dr. Spelman, thought it very strange that Dentists had not found out before this, that they had for many years been making fillings which were not impervious to moisture. Did not think we had been so deceived. Considered it very humiliating if we had been making fillings for several years from which we were unable to exclude the secretions of the mouth. Felt sure that such was not the case. Further discussion on this subject was, on motion, discontinued.

The following resolution was then presented and adopted:

*Resolved*, That when the Association adjourns, it shall adjourn to meet on the second Tuesday of June, 1875, at Put-in-Bay Island.

Dr. Spilman made some very important and interesting remarks in regard to the litigation between the Dentists of Michigan and the Goodyear Dental Vulcanite Co., of Boston.

On motion, the Association adjourned to meet at 1:30 o'clock, p. m.

JUNE 10, 1:30 O'CLOCK, P. M.

Meeting called to order by the Vice-President, R. R. Peebles. No further business of importance appearing, and several members of the Association, wishing to leave at 2 o'clock, p. m. The Association, on motion, adjourned to meet on Put-in-Bay Island on the second Tuesday of June, 1875, at 10 o'clock a. m. L. C. KELSEY, Recording Secretary.

